# Cancer-associated fibroblasts in head and neck tumours: signalling networks and therapeutic implications with insights from vestibular schwannoma

**DOI:** 10.3389/fcell.2026.1927031

**Published:** 2026-09-28

**Authors:** Xiaoli Zhang

**Affiliations:** The Central Hospital Affiliated to Chongqing University of Technology, Chongqing, China

**Keywords:** cancer-associated fibroblasts (CAFs), head and neck squamous cell carcinoma (HNSCC), therapy resistance, tumour microenvironment (TME), vestibular schwannoma (VS)

## Abstract

Cancer-associated fibroblasts (CAFs) are heterogeneous stromal cells that influence extracellular matrix (ECM) organisation, tumour-cell state, immunity, and treatment response in head and neck squamous cell carcinoma (HNSCC). This review synthesises evidence on CAF origins and states and on the transforming growth factor beta (TGF-β), Wnt/β-catenin, PI3K/AKT/mTOR, and Hippo–YAP/TAZ networks that mediate fibroblast interactions with tumour and immune cells. Vestibular schwannoma (VS) is considered only as a hypothesis-generating benign stromal reference: both tumours contain fibroblast and ECM-remodelling compartments, but differ fundamentally in cell of origin, anatomy, oncogenic drivers, immune context, and invasive behaviour. VS stromal fibroblasts are not designated as CAFs unless a study has explicitly applied that term, and cross-tumour observations are not treated as evidence of biological equivalence. Single-cell and spatial studies reveal multiple HNSCC fibroblast states, including myofibroblastic, inflammatory, antigen-presenting and fibroblastic reticular cell (FRC)-like, and immune-regulatory populations, but nomenclature and marker sets remain inconsistent and most inferred functions require perturbational validation. Translational evidence is similarly uneven: broad CAF depletion can be harmful in some non-HNSCC models, whereas pathway- or state-selective modulation is supported mainly by preclinical studies. Early HNSCC trials of TGF-β/programmed death-ligand 1 (PD-L1) blockade and NADPH oxidase 1/4 (NOX1/4) inhibition provide feasibility signals but not definitive efficacy. Current evidence supports biomarker-guided investigation of functionally defined CAF states; stromal observations in VS remain hypothesis-generating and require direct experimental validation.

## Introduction

Head and neck squamous cell carcinoma (HNSCC) is biologically heterogeneous and often clinically aggressive, particularly when locally advanced (Hu et al., 2023; Tsai et al., 2025). The tumour microenvironment (TME) contributes to progression and treatment response, and cancer-associated fibroblasts (CAFs) are a major but non-uniform stromal compartment (Hu et al., 2023; Sahai et al., 2020; Federspiel et al., 2025a; Xie et al., 2025). CAFs can remodel extracellular matrix (ECM), secrete growth factors and cytokines, regulate immune-cell localisation, and alter treatment sensitivity; however, these functions vary across fibroblast states and experimental contexts (Hu et al., 2023; Sahai et al., 2020; Takahashi et al., 2026; Mao et al., 2021). Interpretation depends on distinguishing direct HNSCC evidence from findings extrapolated from other tumour types.

Benign stromal tumours are not interchangeable with HNSCC. Vestibular schwannoma (VS) arises from Schwann-lineage cells of the vestibular nerve and is usually non-metastatic, whereas HNSCC arises from mucosal epithelium in a carcinogen- and/or human papillomavirus (HPV)-associated field and can invade and metastasise. VS studies nevertheless identify fibroblast, ECM-remodelling, and immune–stromal compartments, together with candidate paracrine interactions, that can be interrogated using single-cell and spatial methods (Sung and Lee, 2024; Barrett et al., 2024; Gonzalez Castro et al., 2025; Huo et al., 2024; Xu et al., 2022). These shared analytical domains provide a limited rationale for comparison, whereas differences in cell lineage, anatomical constraints, oncogenic drivers, ECM composition, immune exposure, and treatment context preclude direct equivalence. The studies reviewed here do not include a matched comparison of HNSCC CAFs and VS fibroblasts. Accordingly, VS is treated as a hypothesis-generating stromal reference intended to define experimentally testable questions, not to explain the benign behaviour of VS or to model HNSCC malignancy. The term “VS fibroblasts” is used throughout; “CAF” or “CAF-like” is reserved for instances in which the cited VS study used that terminology, without implying equivalence to CAFs in malignant tumours.

The review evaluates fibroblast origin and heterogeneity together with TGF-β, Wnt/β-catenin, PI3K/AKT/mTOR, and Hippo–YAP/TAZ signalling, tumour behaviour, immune regulation, therapy resistance, and stromal therapeutic strategies. Evidence is classified as HNSCC-specific, VS-specific, or derived from other tumour types; transcriptomic associations and ligand–receptor predictions are distinguished from functional perturbation data, and preclinical findings are distinguished from clinical evidence.

## Origin and heterogeneity of CAFs

CAFs arise from multiple cellular sources and activation trajectories rather than a single lineage (Sahai et al., 2020; Mao et al., 2021; LeBleu and Kalluri, 2018; Kalluri and Zeisberg, 2006). In carcinomas, resident tissue fibroblasts are a major source, while mesenchymal stromal cells, perivascular cells, and endothelial-derived mesenchymal cells can contribute in model- and tissue-dependent ways (LeBleu and Kalluri, 2018; Frisbie et al., 2022; Han et al., 2025; Guo and Xu, 2024). Partial epithelial–mesenchymal transition can produce tumour cells with mesenchymal features, but does not demonstrate their conversion into CAFs. Tumour-derived TGF-β, CXCL12, interleukins, and mechanical cues can recruit or activate stromal fibroblasts, echoing but not reproducing the wound-healing programme (Sahai et al., 2020; Dvorak, 2019).

CAF populations may also receive contributions from sources beyond local fibroblasts. In model-dependent settings, bone-marrow-derived mesenchymal stromal cells can migrate to tumours and adopt fibroblast-like programmes (Frisbie et al., 2022). Some carcinoma cells undergo partial epithelial–mesenchymal transition and acquire mesenchymal features, but this is not equivalent to proven conversion into CAFs (LeBleu and Kalluri, 2018). Endothelial-to-mesenchymal transition, perivascular cells, and adipocyte-derived stromal cells have been proposed as additional sources (Han et al., 2025; Guo and Xu, 2024). These routes are not mutually exclusive, and their quantitative contribution in human HNSCC remains uncertain (Figure 1a) (Sahai et al., 2020; LeBleu and Kalluri, 2018).

**FIGURE 1:**
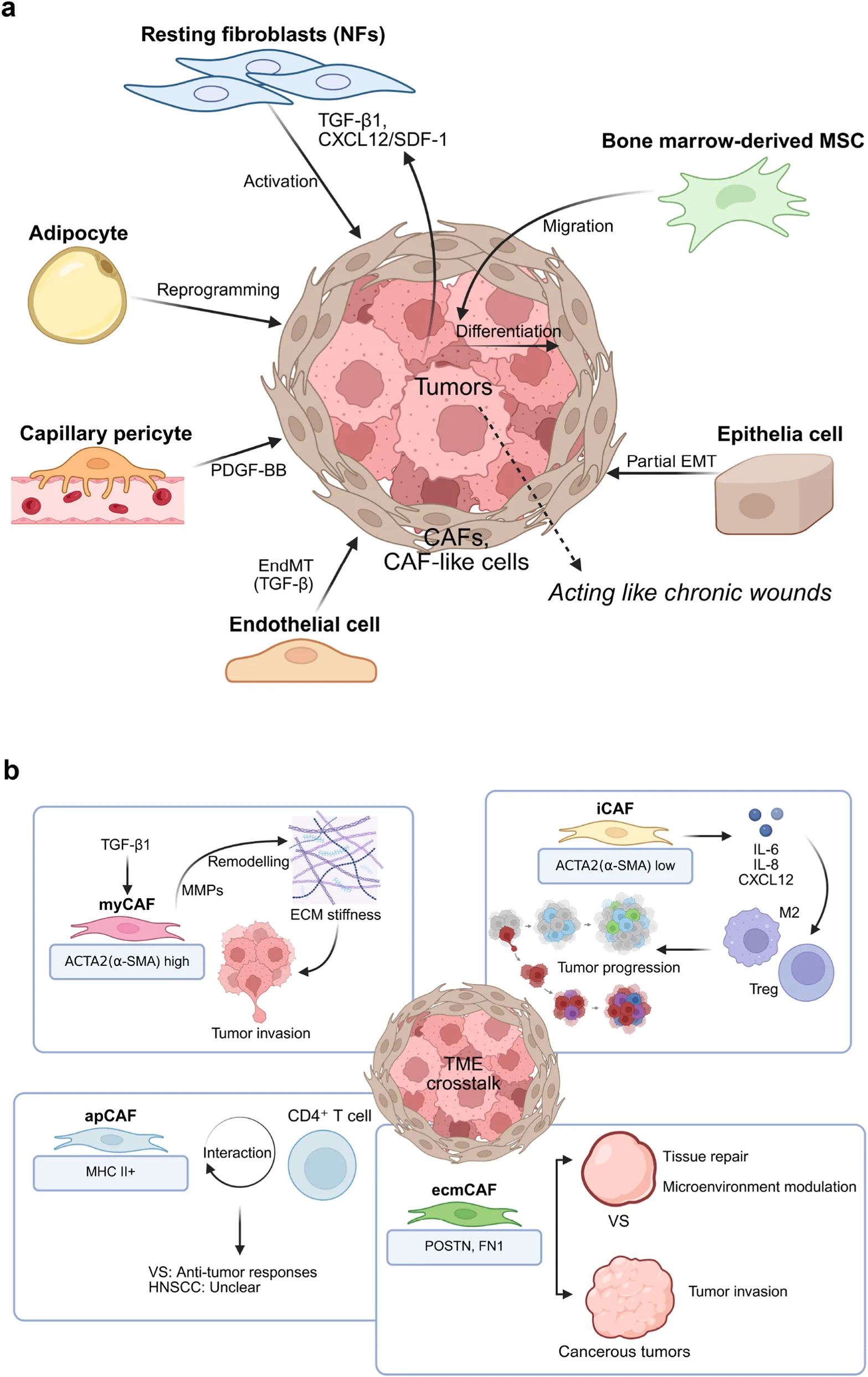
Origins and heterogeneity of CAFs. **(a)** Proposed cellular origins of CAFs, including resident fibroblasts, mesenchymal stem cells, endothelial cells, pericytes, adipocytes, and epithelial cells undergoing partial EMT. **(b)** Major CAF states and representative programmes: myofibroblastic, inflammatory, antigen-presenting-like, and ECM-rich. VS clusters are shown as transcriptional observations rather than functional equivalents of HNSCC CAF states. Created in BioRender. Zhang, X. (2026) https://BioRender.com/0mhsfwx.

Single-cell studies have resolved substantial fibroblast diversity in HNSCC and schwannoma, but their datasets are not directly comparable. Puram et al. profiled approximately 6,000 cells from 18 HNSCC specimens and linked a partial epithelial–mesenchymal transition programme at the invasive edge to stromal interactions (Puram et al., 2017). Subsequent HNSCC studies identify myofibroblastic, inflammatory, immune-regulatory, and fibroblastic-reticular-cell-like states, with spatial position and HPV/immune context influencing their abundance (Jenkins et al., 2025; Furudate et al., 2025; Qiu et al., 2025; Li C. et al., 2024; Obra et al., 2022). By contrast, VS studies have profiled small cohorts or re-analysed published datasets: Huo et al. analysed seven VS and two normal nerves; Sung and Lee re-analysed three VS; and a broader schwannoma atlas sampled 22 cranial and peripheral tumours across genetic backgrounds (Sung and Lee, 2024; Barrett et al., 2024; Gonzalez Castro et al., 2025; Huo et al., 2024; Xu et al., 2022). VS datasets reveal heterogeneity and nominate candidate cell–cell interactions, but do not establish one-to-one correspondence between HNSCC CAF subsets and VS fibroblasts.

Myofibroblastic CAFs (myCAFs) are generally enriched for ACTA2/alpha-smooth muscle actin (α-SMA), contractile genes, collagen and fibronectin production, and ECM organisation, often near tumour nests (Elyada et al., 2019; Biffi and Tuveson, 2021). TGF-β and mechanical tension can stabilise this programme. In oral squamous cell carcinoma, spatial analysis placed a myCAF population at the invasive front and linked ECM–CD44 interactions with lymph-node metastasis (Furudate et al., 2025). Nevertheless, α-SMA is neither exclusive to CAFs nor sufficient to define a tumour-promoting function; pericytes and wound-associated myofibroblasts can share this marker.

Inflammatory CAFs (iCAFs) are characterised by cytokine and chemokine programmes that may include IL-6, IL-8, CXCL12, or IL-11, usually with lower contractile-gene expression (Elyada et al., 2019; Biffi and Tuveson, 2021). In HNSCC, Jenkins et al. identified six fibroblast subtypes across HPV-negative, HPV-positive, and non-malignant tissues, including IL11-positive inflammatory and CCL19-positive fibroblastic-reticular-cell-like populations (Jenkins et al., 2025). The latter were associated with immune-hot niches, tertiary lymphoid structures, and favourable immunotherapy response. The reported immune-hot association of CCL19-positive FRC-like fibroblasts illustrates why inflammatory and immune-associated fibroblast states cannot be assumed to be uniformly immunosuppressive; association, spatial colocalisation, and *ex vivo* function represent distinct levels of evidence.

Antigen-presenting CAFs (apCAFs) express major histocompatibility complex (MHC) class II and antigen-processing genes, but their consequences depend on co-stimulation, location, and immune context. They were first functionally characterised in pancreatic cancer (Elyada et al., 2019). MHC-II-expressing stromal or tumour-associated populations have also been reported in schwannoma datasets (Sung and Lee, 2024; Gonzalez Castro et al., 2025; Dong et al., 2025), but this does not show that they are equivalent to pancreatic apCAFs or that they activate versus tolerise CD4^+^ T cells. Their identity and function in HNSCC remain incompletely resolved.

Additional labels—including ECM-rich, cycling, fibroblast activation protein (FAP)-positive, leucine-rich repeat-containing protein 15 (LRRC15)-positive, MHC-I-high/Galectin-9-positive, and FRC-like fibroblasts—capture partly overlapping states (Li C. et al., 2024; Cords et al., 2024; Krishnamurty et al., 2022; Dominguez et al., 2020). A pan-cancer LRRC15-positive myofibroblast programme is TGF-β-associated and correlated with poor checkpoint-blockade response, while selective depletion enhanced anti-PD-L1 activity in pancreatic mouse models (Krishnamurty et al., 2022; Dominguez et al., 2020). In HNSCC, an MHC-I-high/Galectin-9-positive CAF subset spatially restricted CD8^+^ T-cell infiltration and function (Li C. et al., 2024), whereas immunostimulatory HNCAF-0/HNCAF-3 populations predicted response in a small discovery cohort (Obra et al., 2022). These apparently conflicting observations likely reflect true state diversity plus differences in sampling, annotation, and assay design (Figure 1b).

No universal marker panel yet separates tumour-promoting from tumour-restraining fibroblasts, and the same marker can occur in several states or in normal stromal cells (Sahai et al., 2020; Biffi and Tuveson, 2021; Cords et al., 2024). Most single-cell studies are cross-sectional, contain limited patient numbers, and infer function from transcription or ligand–receptor databases. Within this evidence hierarchy, transcriptomic association defines a candidate state; spatial colocalisation supports proximity but not signalling direction; ligand–receptor analysis generates hypotheses; perturbation or co-culture provides model-specific functional evidence; and trial outcomes constitute clinical evidence. Lineage tracing and treatment-linked longitudinal sampling remain necessary before a transcriptomic cluster can be treated as a therapeutic target.

Classifications that integrate fibroblast state, spatial location, and function are more clinically informative than fixed cell categories. Key unresolved questions are whether HNSCC fibroblast states are stable or interconvertible, which states cause rather than accompany immune exclusion and resistance, how HPV status and treatment reshape them, and whether any VS fibroblast programme can serve as a neutral comparator after controlling for tissue origin and anatomy (Table 1).

**TABLE 1:** Fibroblast states across HNSCC and VS: evidence and limitations.

Fibroblast state	Key features	Evidence in HNSCC	VS or schwannoma observation	Key limitation
myCAF	ACTA2/α-SMA, contractility, collagen/ECM	Invasive-front myCAFs; ECM–CD44 association with nodal metastasis (Furudate et al., 2025)	Contractile/ECM-rich clusters reported (Sung and Lee, 2024; Gonzalez Castro et al., 2025)	α-SMA is not specific; state can be reversible
iCAF	IL-6/IL-8/CXCL12/IL-11 programmes	IL-11-positive inflammatory states; immune functions remain incompletely resolved (Jenkins et al., 2025)	Inflammatory clusters reported computationally (Sung and Lee, 2024)	Different cytokine sets are grouped under one label
apCAF/MHC-II+	MHC-II and antigen-processing genes	Identity and function incompletely resolved	MHC-II-rich programmes observed (Gonzalez Castro et al., 2025; Dong et al., 2025)	Expression does not prove productive antigen presentation
FRC-like/immunostimulatory	CCL19/CXCL13; lymphoid organisation	Associated with immune-hot niches/TLS and response (Jenkins et al., 2025; Obra et al., 2022)	Candidate immune-associated fibroblasts (Sung and Lee, 2024; Gonzalez Castro et al., 2025)	Small cohorts; prospective validation absent
Immune-excluding	MHC-I-high/Galectin-9; matrix/TGF-β-linked	Spatial restriction and CD8 dysfunction (Li et al., 2024a)	No validated equivalent	Marker overlap and tumour-context dependence
LRRC15+ myofibroblast	TGF-β-associated myofibroblast programme	Pan-cancer association; HNSCC-specific role unresolved (Krishnamurty et al., 2022; Dominguez et al., 2020)	Not validated	Functional depletion evidence is mainly pancreatic mouse data

## CAF–tumour signalling pathways

CAFs communicate with tumour and immune cells through intersecting TGF-β, Wnt/β-catenin, PI3K/AKT/mTOR, and Hippo–YAP/TAZ networks (Figures 2a–d) (Hu et al., 2023; Sahai et al., 2020; Athavale et al., 2024; Wu et al., 2021). These pathways are not CAF-specific and can operate in tumour, immune, endothelial, and Schwann-lineage cells. Pathway activity in VS does not establish an identical cellular source or biological consequence in HNSCC. The supporting evidence and its interpretive limits are stratified by disease setting (Table 2).

**FIGURE 2:**
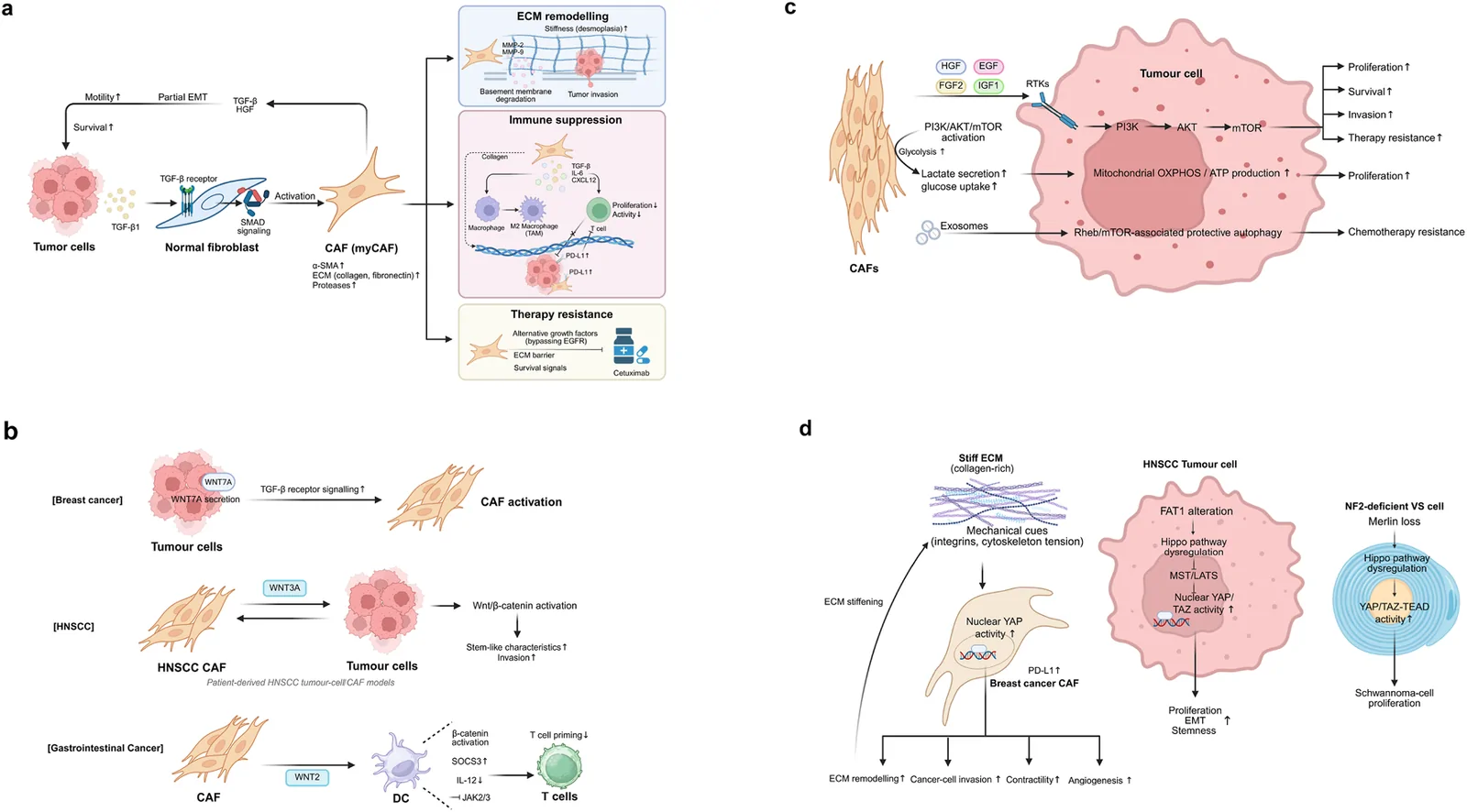
CAF–tumour signalling pathways. **(a)** TGF-β1–SMAD signalling promotes myofibroblastic CAF activation and downstream matrix remodelling, immune suppression, and cetuximab resistance in HNSCC and related carcinoma models. **(b)** Model-specific Wnt interactions involving tumour-cell-derived WNT7A in breast cancer, WNT3A-responsive tumour–stromal signalling in HNSCC, and WNT2-mediated dendritic-cell suppression in gastrointestinal cancer. **(c)** Growth-factor, metabolic, and exosomal signalling can activate tumour-cell PI3K/AKT/mTOR, mitochondrial metabolism, or Rheb/mTOR-associated autophagy across head and neck cancer models. **(d)** Hippo–YAP/TAZ signalling is shown separately in breast-cancer CAF mechanotransduction, FAT1-altered HNSCC tumour cells, and NF2-deficient schwannoma cells; these mechanisms are not presented as equivalent. Created in BioRender. Zhang, X. (2026) https://BioRender.com/uaijav7.

**TABLE 2:** Pathway evidence in HNSCC and related head and neck tumour models, VS observations, and interpretive limitations.

Axis	HNSCC and related head and neck models	VS or schwannoma evidence	Interpretive limitation
TGF-β	CAF activation, invasion, cetuximab resistance; preclinical blockade (Wang et al., 2022; Yegodayev et al., 2020; Vigneron et al., 2026)	NF2-SKOR2 model showed reduced SMAD2/3 response (Tang et al., 2025)	No proof of increased TGF-β receptor expression or VS-fibroblast-driven fibrosis
Wnt/β-catenin	Patient-derived HNSCC tumour-cell/CAF models: WNT3A activates both compartments; CAF co-culture enhances stem-like characteristics and invasion (Le et al., 2019)	Predicted SPP1–CD44 and other interactions (Xu et al., 2022; Dong et al., 2025)	Ligand–receptor inference is not functional equivalence
PI3K/AKT/mTOR	OSCC: ITGB2–PI3K/AKT/mTOR-driven CAF lactate release; NPC: exosomal Rheb/mTOR–autophagy and cisplatin resistance (Wu et al., 2021; Zhang et al., 2020; Meng et al., 2024)	Merlin loss activates mTORC1 in tumour cells (James et al., 2009)	Tumour-cell signalling does not establish fibroblast activity
Hippo–YAP/TAZ	HNSCC stromal nuclear YAP; HNCAF YAP/TAZ perturbation in organotypic invasion models (Athavale et al., 2024; Calvo et al., 2013)	Merlin loss and stiffness provide plausible tumour-cell links (Zhang et al., 2025a; Petrilli and Fernandez-Valle, 2016)	VS-fibroblast YAP/TAZ function remains unproven

## TGF-β pathway

Transforming growth factor beta (TGF-β) is a principal regulator of fibroblast activation in wound healing, fibrosis, and cancer-associated stroma (Biffi and Tuveson, 2021). Signalling through TGF-β1/2 and SMAD proteins can induce α-SMA-positive, extracellular matrix-producing myofibroblast programmes (Hu et al., 2023; Biffi and Tuveson, 2021). In HNSCC, reviews identify tumour-derived TGF-β as a recurrent candidate driver of fibroblast activation (Mou et al., 2023). However, the underlying studies use heterogeneous fibroblast sources and model systems and do not establish a uniform TGF-β-dependent CAF state across patients (Mou et al., 2023). Other soluble cues can converge on or oppose this programme: experiments in other tumour types identified epigenetically distinct leukaemia inhibitory factor (LIF)- and TGF-β-dependent routes to CAF activation (Albrengues et al., 2015), whereas TGF-β1 and fibroblast growth factor 2 induced partly divergent CAF transcriptional states (Bordignon et al., 2019). The distinct LIF-, TGF-β-, and FGF2-associated transcriptional responses support stimulus-dependent fibroblast plasticity rather than a single canonical activation pathway.

Bidirectional TGF-β signalling can connect fibroblast and tumour-cell phenotypes. HNSCC reviews describe CAF-derived TGF-β and hepatocyte growth factor (HGF) as candidate drivers of partial epithelial–mesenchymal transition, motility, and apoptosis resistance (Mou et al., 2023). Across experimental models, TGF-β can also increase fibroblast expression of matrix-remodelling proteases, including matrix metalloproteinase (MMP)-2 and MMP-9 (Xie et al., 2025). In an oral squamous cell carcinoma model, halofuginone-mediated suppression of CAF activation decreased MMP2 expression and nodal metastasis (Wang et al., 2022). Across these models, TGF-β-associated fibroblast activation is linked to extracellular-matrix remodelling and invasion, but the available experiments do not establish a single linear mechanism across HNSCC.

CAF-derived TGF-β can shape immune exclusion, but the evidence is strongly tumour- and model-dependent. In a non-HNSCC model, stromal TGF-β signalling was associated with collagen-rich T-cell exclusion and resistance to PD-L1 blockade (Mariathasan et al., 2018). This supports a mechanism in another tumour type, not direct proof that CAF-derived TGF-β is the dominant cause of immunotherapy failure in HNSCC. TGF-β, YAP/TAZ, and checkpoint-ligand programmes may intersect in the TME, but the cited studies do not establish a universal TGF-β-driven increase in PD-L1 across both HNSCC tumour cells and CAFs (Kim and Nam, 2025; Tauriello et al., 2018).

Preclinical HNSCC models also implicate TGF-β-activated CAFs in resistance to cetuximab, an epidermal growth factor receptor (EGFR)-targeted antibody (Hu et al., 2023). Stromal TGF-β inhibition improved cetuximab response in experimental models (Yegodayev et al., 2020), supporting this pathway as a candidate combination target. Clinical efficacy remains unestablished, and broad TGF-β inhibition may affect tumour cells, immune cells, fibroblasts, and normal tissue repair (Figure 2a).

Current evidence does not support the claim that Merlin loss both suppresses and sensitises TGF-β signalling in VS. In an NF2-associated model of human Schwann cells derived from induced pluripotent stem cells, pathogenic NF2 variants promoted nuclear SKOR2 accumulation and were associated with reduced SMAD2/3 phosphorylation and lower TGF-β reporter activity; the study did not demonstrate increased TGF-β-receptor expression, generalised growth-factor hypersensitivity, or fibroblast-driven fibrosis in patient VS (Tang et al., 2025). TGF-β ligands and fibrotic features may be present in VS tissue, but their cellular source, direction of pathway activity, and causal significance remain unresolved. The better-established effects of Merlin loss involve Hippo–YAP/TAZ and mTORC1 signalling (James et al., 2009; Petrilli and Fernandez-Valle, 2016). Available VS data do not support TGF-β inhibition or tranilast as a therapeutic strategy; evaluation would require direct testing in VS fibroblast–Schwann-cell models and *in vivo* validation.

## Wnt/β-catenin pathway

Wnt/β-catenin signalling participates in bidirectional tumour–fibroblast communication, but its cellular source and functional consequence vary across tumour types.

The cellular direction of tumour–fibroblast Wnt signalling differs among tumour models. In breast-cancer models, tumour-cell-derived WNT7A enhanced TGF-β-receptor signalling in fibroblasts and promoted a CAF phenotype (Avgustinova et al., 2016); this is evidence from breast-cancer models rather than proof of the same recruitment mechanism in HNSCC. In matched patient-derived HNSCC tumour-cell/CAF models, WNT3A activated Wnt signalling in both compartments, and CAF co-culture increased cancer-cell stem-like characteristics and invasion. Pharmacological Wnt-pathway inhibition reduced signalling at the tumour–stromal interface and suppressed the growth of patient-derived xenografts (Le et al., 2019).

CAFs can also secrete Wnt ligands that regulate tumour or immune cells. WNT2-positive CAFs suppressed dendritic-cell maturation through β-catenin–SOCS3 signalling and impaired antitumour T-cell priming in gastrointestinal cancer models; WNT2 blockade restored dendritic-cell function (Huang et al., 2022). An equivalent WNT2-positive CAF programme or therapeutic effect has not been established in HNSCC.

CAF-derived Wnt-modulatory signals can support stem-like tumour-cell states in several cancers. In HNSCC, matched tumour-cell/CAF models support regulation of Wnt activity at the tumour–stromal interface (Le et al., 2019). In VS, ligand–receptor analyses have nominated SPP1–CD44 and other fibroblast–Schwann-cell interactions (Xu et al., 2022; Dong et al., 2025), but computational interaction scores do not prove pathway activation or a cancer-stem-cell niche. The VS observations generate hypotheses for perturbation experiments but do not demonstrate a shared malignant mechanism (Figure 2b).

## PI3K/AKT/mTOR pathway

The PI3K/AKT/mTOR pathway regulates cell growth, metabolism, and survival in HNSCC and can be modulated by stromal growth factors and matrix signals (Wu et al., 2021; Hsieh et al., 2019; Martinez-Outschoorn et al., 2017). Because pathway activation is common in both tumour and stromal compartments, mechanistic interpretation depends on identifying the responding cell type.

CAF-derived HGF, EGF-family ligands, FGF2, and IGF1 can activate receptor tyrosine kinases and downstream PI3K/AKT signalling in tumour cells (Wu et al., 2021). Such bypass signalling may promote proliferation, invasion, and treatment resistance, but the relative contribution of each ligand varies by tumour and therapy. Evidence from lung adenocarcinoma is insufficient to assign specific anti-apoptotic programmes to HNSCC without direct validation.

PI3K/AKT/mTOR signalling can also shape fibroblast metabolism. An oral squamous cell carcinoma study identified an ITGB2-high CAF subset associated with PI3K/AKT/mTOR pathway activation and metabolic reprogramming, including increased glucose uptake and lactate production (Zhang et al., 2020). The ITGB2-high state provides disease-relevant evidence for a metabolic CAF state, but it does not establish that all HNSCC CAFs share this programme or that PI3K/AKT/mTOR inhibition would selectively disrupt the stromal compartment.

Primary experimental evidence from nasopharyngeal carcinoma (NPC) indicates that CAF-derived exosomes can enhance cisplatin resistance by promoting autophagy in tumour cells through Rheb/mTOR signalling (Meng et al., 2024). The study included *in vitro* analyses and a xenograft model, supporting a stromal exosome–autophagy mechanism in NPC. It does not establish that the same pathway operates in HNSCC, and the active exosomal cargo, prevalence across fibroblast states, and contribution in patients require independent validation.

In benign VS, Merlin loss can activate mTORC1 in tumour cells (James et al., 2009). Separately, a 2025 study associated ECM remodelling and increased stiffness with FAK activation; matrix stiffness promoted cytoskeletal remodelling, proliferation, and metalloproteinase expression in VS cells, while FAK inhibition diminished these effects (Zhang et al., 2025a). The study did not establish that fibroblast-derived ECM is the source of stiffness or that FAK acts through PI3K/AKT in VS. mTORC1 activation and stiffness–FAK signalling are parallel tumour-cell observations rather than a demonstrated fibroblast–PI3K feedback loop (Figure 2c).

## Hippo–YAP/TAZ pathway

The Hippo pathway and its YAP/TAZ transcriptional co-activators respond to cell density, matrix stiffness, and cytoskeletal tension (Athavale et al., 2024; Zan et al., 2016; Panciera et al., 2017). MST1/2–LATS1/2 signalling restrains YAP/TAZ under homeostatic conditions, whereas oncogenic alteration or sustained mechanical input can favour nuclear YAP/TAZ activity. Because these mechanisms were established across multiple tissues and model systems, assigning them to HNSCC CAF subsets requires cell-type-resolved evidence rather than inference from unrelated organ-specific knockout phenotypes.

YAP-dependent mechanotransduction can help maintain an activated fibroblast phenotype. In breast-cancer-derived fibroblasts, YAP was required for matrix stiffening, cancer-cell invasion, and angiogenesis; matrix stiffening further enhanced fibroblast YAP activation, creating a self-reinforcing loop (Calvo et al., 2013). This primary study did not test HNSCC or VS fibroblasts, so the same circuit in those diseases remains to be demonstrated.

FAT1 can assemble the Hippo signalosome and restrain YAP activity, providing a tumour-cell mechanism relevant to FAT1-altered HNSCC (Martin et al., 2018). Reviews also describe YAP/TAZ-dependent stromal, vascular, and immune programmes across cancer models (Athavale et al., 2024; Kim and Nam, 2025), but they do not establish that stromal YAP is equally important as tumour-cell YAP in HNSCC or that YAP-driven PD-L1, VEGF, and angiopoietin production is a single validated HNSCC CAF mechanism.

Matrix stiffness can activate YAP-dependent fibroblast programmes in experimental cancer models (Calvo et al., 2013). In HNSCC, however, direct perturbational evidence assigning this response to defined CAF states remains limited. The VS stiffness–FAK study (Zhang et al., 2025a) did not measure YAP/TAZ activation and therefore provides no direct evidence for a stiffness–FAK–YAP feedback loop in VS fibroblasts.

In VS, Merlin loss provides a plausible link to Hippo–YAP/TAZ dysregulation in neoplastic Schwann cells (Petrilli and Fernandez-Valle, 2016). However, evidence that YAP/TAZ is activated specifically in VS fibroblasts, or that fibroblast YAP/TAZ causes tumour fibrosis or Schwann-cell proliferation, remains insufficient. Matrix stiffness and FAK signalling correlate with VS progression (Zhang et al., 2025a), but the cellular direction of the proposed stiffness–YAP feedback requires spatial phosphoproteomic or perturbational validation. Shared pathway components do not imply shared malignant behaviour.

YAP/TAZ modulation is a preclinical stromal strategy rather than an established HNSCC therapy. Experimental studies indicate that fibroblast YAP/TAZ activity regulates matrix remodelling and cancer-cell invasion; in a lung squamous carcinoma model, mechanically induced fibroblast YAP signalling also promoted myeloid recruitment and reduced CD8^+^ T-cell infiltration (Calvo et al., 2013; Cai et al., 2026). Verteporfin studies in HNSCC predominantly address tumour-cell YAP1 (Liu et al., 2019), and verteporfin is not a selective clinical YAP/TAZ inhibitor. Translation will require CAF-state biomarkers, on-target pharmacodynamic measurements, and attention to YAP/TAZ functions in tissue repair and normal homeostasis.

Experimental cancer models link mechanical cues to Hippo–YAP/TAZ-dependent fibroblast transcriptional programmes (Panciera et al., 2017), but the contribution to aggressiveness and treatment resistance remains context dependent. Simultaneously affecting tumour and stromal YAP/TAZ is not necessarily advantageous because the pathway also supports normal tissue homeostasis and repair. Any therapeutic application would require cell-type-resolved pharmacodynamic evidence and a defined therapeutic window (Figure 2D).

## CAF influence on tumour behaviour

CAFs regulate tumour growth, invasion, and immunity through state-dependent mechanisms within the HNSCC microenvironment. Their immune effects range from exclusion to immune support; comparison with VS is interpretable only when the relevant cell types and readouts are resolved (Spiljak et al., 2025).

CAFs can enhance HNSCC-cell proliferation and survival through growth factors, chemokines, and metabolic exchange, but the responsible mediators vary between models (Li C. et al., 2024; Wu et al., 2021; Zhang et al., 2024; Magan et al., 2020). In HNSCC models, HGF secreted by tumour-derived fibroblasts activated MET in tumour cells and promoted proliferation and invasion; HGF neutralisation inhibited fibroblast-induced invasion, whereas MET inhibition reduced xenograft growth (Knowles et al., 2009). Lactate transfer can support tumour metabolism (Figure 3a). Patient-derived HNSCC CAFs increased tumour-cell growth or treatment resistance in three-dimensional co-culture systems (Magan et al., 2020), although such assays cannot reproduce the full immune and vascular microenvironment. VS fibroblasts also participate in paracrine networks, and VS tissue can secrete factors including FGF2 (Xu et al., 2022; Baruah et al., 2023; Dilwali et al., 2013). Available studies have not shown that VS fibroblasts produce quantitatively lower EGF/HGF than HNSCC CAFs, determine a growth ceiling, or explain the benign behaviour of VS.

**FIGURE 3:**
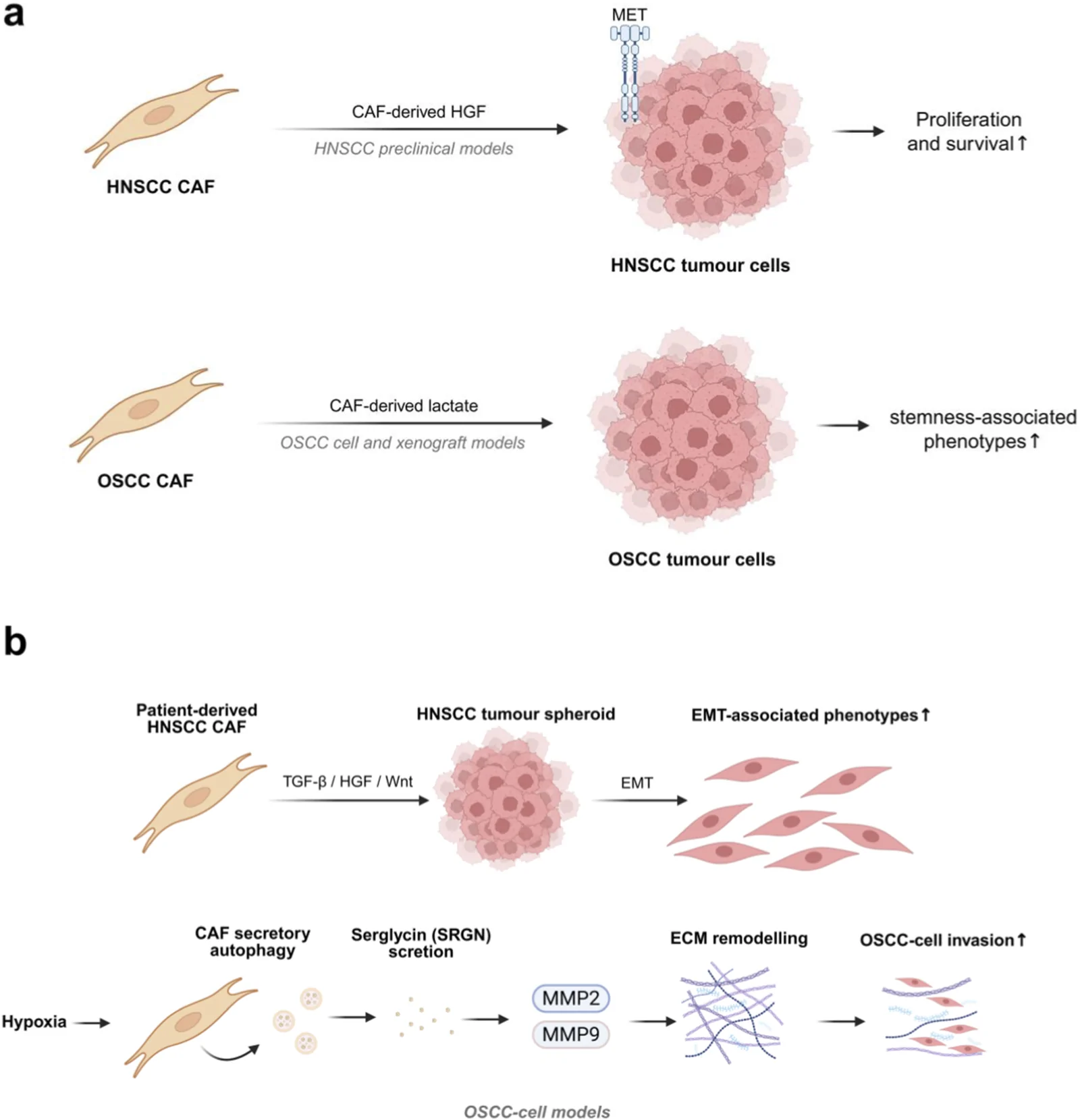
Pro-tumoural functions of CAFs in tumour progression. **(a)** HNSCC CAF-derived HGF activates tumour-cell MET and supports proliferation and survival. In OSCC models, CAF-derived lactate promotes stemness-associated phenotypes through the DLG5–CUL3–MST1–YAP1 axis (Zhang et al., 2024).**(b)** Patient-derived HNSCC CAFs promote EMT-associated phenotypes in three-dimensional co-culture. In OSCC models, hypoxia-induced CAF secretory autophagy promotes serglycin release, MMP2/MMP9-associated matrix remodelling, and invasion. Created in BioRender. Zhang, X. (2026) https://BioRender.com/eddn2c7.

### Promoting invasion and metastasis

CAFs can promote invasion through soluble signals and mechanical remodelling of the extracellular matrix. TGF-β, HGF, and Wnt-family ligands can induce epithelial–mesenchymal plasticity in tumour cells, although the relative contribution of each ligand is model-dependent. In a patient-derived HNSCC three-dimensional co-culture model, CAF exposure increased an EMT-associated tumour-cell phenotype (Magan et al., 2020). In HNSCC, a partial epithelial–mesenchymal-transition programme at the invasive edge was associated with stromal interactions and single-cell invasion, but was not assigned specifically to CAF-derived TGF-β or proteases (Park et al., 2025). In oral squamous cell carcinoma, hypoxia-induced secretory autophagy in CAFs promoted serglycin release; extracellular serglycin interacted with MMP2/MMP9 and enhanced extracellular-matrix remodelling and invasion in experimental models (Zhang et al., 2025b).

CAFs physically reshape the ECM by depositing collagen, fibronectin, and other proteins and by generating forces that align fibres. A dense collagenous stroma may impede movement when highly compacted yet provide aligned migration tracks when organised by CAF tension. CAF-associated matrix stiffness can activate integrin–FAK signalling in tumour cells and promote invadopodia and motility (Knipper et al., 2023). In VS, tissue stiffness was associated with FAK activation and Schwann-cell proliferation (Zhang et al., 2025a), but the cellular source and direction of this mechanical relationship remain incompletely resolved. Periostin expression in OSCC has been associated with epithelial–mesenchymal transition, invasion, and lymph-node metastasis (Won et al., 2025; Zhang et al., 2025b) (Figure 3b).

CAFs may also support dissemination through lymphangiogenic and pre-metastatic-niche signals, but much of the mechanistic evidence derives from non-HNSCC models (Xie et al., 2025; Bhowmick et al., 2004; Kaplan et al., 2005; Cadamuro et al., 2019). In HNSCC, lymph-node spread is clinically central and spatial myCAF–tumour interactions at the invasive front are associated with nodal metastasis (Furudate et al., 2025). Directly assigning VEGF-C/VEGF-D, CCL21, or a lymph-node niche to a specific HNSCC CAF state requires cell-type-resolved and functional validation.

VS is usually circumscribed and non-metastatic, and VS tissue can express MMP-2 and MMP-9, particularly in cystic lesions (Moller et al., 2010; Moon et al., 2007). However, current data do not show that VS fibroblasts create a protective capsule, restrict invasion, or convert MMP activity exclusively into cyst formation. Conversely, the absence of metastasis cannot be attributed to a fibroblast programme without controlling for Schwann-cell lineage, NF2 biology, anatomical environment, and tumour genomics. Resolving whether distinct stromal programmes are associated with local tissue support or invasion requires matched spatial and functional studies that do not presuppose an intrinsically protective VS fibroblast state.

Fibroblast-dependent vascular and lymphatic effects differ across tumour models. In invasive breast-carcinoma models, stromal fibroblast-derived SDF-1/CXCL12 supported angiogenesis and tumour growth (Orimo et al., 2005); this does not establish the same quantitative dependency in HNSCC or NPC. In an inflammation-associated mouse cancer model, NF-κB-dependent CAF programmes supported tumour-promoting inflammation (Erez et al., 2010). In cholangiocarcinoma, navitoclax-mediated CAF reduction decreased tumour lymphangiogenesis and nodal metastasis (Cadamuro et al., 2019). Evidence that a defined HNSCC CAF state directly recruits pericytes or drives VEGF-C/VEGF-D-dependent lymphangiogenesis remains limited, and these findings from other tumour types do not establish that broad CAF depletion will reduce metastasis in HNSCC.

Angiogenesis contributes to VS growth, and bevacizumab can improve hearing or reduce tumour volume in some patients with NF2-related VS (Plotkin et al., 2009; Screnci et al., 2024). This clinical response supports a role for VEGF signalling in a subset of tumours but does not identify fibroblasts as the principal VEGF source. Single-cell datasets nominate vascular and stromal interactions, yet fibroblast-specific angiogenic function and the relevance of lymphatic programmes in VS remain unresolved (Barrett et al., 2024; Gonzalez Castro et al., 2025; Huo et al., 2024; Xu et al., 2022).

### CAF-mediated immune evasion

Fibroblasts can shape immune composition through cytokines, chemokines, matrix, and metabolic signals, although the direction and magnitude of these effects depend on fibroblast state and tumour type (Kalluri, 2016). TGF-β-, IL-6-, IL-8-, prostaglandin-, and NF-κB-associated programmes have been linked to myeloid or lymphocyte regulation across experimental cancers. However, the cited NF-κB-dependent inflammatory CAF programme was demonstrated in a mouse model of inflammation-associated skin carcinogenesis (Erez et al., 2010), rather than in HNSCC. The skin-carcinogenesis model does not support inference of a uniform secretory profile or dominant immune-regulatory mechanism across HNSCC CAF states.

Chemokine-mediated recruitment and retention: FAP-positive stromal-cell-derived CXCL12 restricted T-cell access and limited PD-L1 blockade in a pancreatic cancer model (Feig et al., 2013). Reviews describe additional CCL2-, CCL5-, monocyte-, and suppressor-cell interactions across tumours (Turley et al., 2015), but their relevance to HNSCC remains contingent on cell-type-resolved functional evidence.

CAF-associated ECM can restrict lymphocyte access to tumour islets in experimental models (Mariathasan et al., 2018). FAP-expressing stromal cells can suppress antitumour immunity *in vivo* (Kraman et al., 2010), but that study did not establish PD-L2 or YAP-dependent PD-L1 as universal CAF mechanisms. Likewise, TGF-β blockade relieved stromal T-cell exclusion in a non-HNSCC model (Mariathasan et al., 2018), leaving the relative contribution of CAF-secreted TGF-β or IL-10 in OSCC unresolved.

The functional consequences of antigen presentation by antigen-presenting CAFs depend on co-stimulatory capacity, anatomical location, and the immune milieu. MHC class II expression indicates antigen-processing capacity, but productive CD4^+^ T-cell activation also requires appropriate peptide presentation, co-stimulation, and spatial contact. In pancreatic models, antigen-presenting CAFs promoted regulatory or tolerogenic CD4^+^ T-cell responses (Elyada et al., 2019); equivalent function has not been established in HNSCC.

Fibroblast-rich HNSCC can exhibit immune-excluded phenotypes, but the relationship is not uniform. An MHC-I-high/Galectin-9-positive CAF subset restricted CD8^+^ T-cell infiltration and function in spatial and single-cell analyses (Li C. et al., 2024). Conversely, a four-patient paired single-cell discovery cohort and *ex vivo* experiments identified HNCAF-0/HNCAF-3 populations associated with immunostimulation and checkpoint-inhibitor response (Obra et al., 2022), while CCL19-positive FRC-like fibroblasts were enriched in immune-hot, tertiary-lymphoid-structure-containing HNSCC (Jenkins et al., 2025). The opposing immune associations are compatible with state-dependent fibroblast effects, but modest cohorts, non-identical taxonomies, and limited prospective validation preclude a universal predictive CAF signature.

VS also contains immune and stromal populations, but available studies do not justify describing its fibroblasts as uniformly permissive to immune infiltration or intrinsically less suppressive than HNSCC CAFs. Single-cell and spatial studies report variable macrophage, T-cell, antigen-presentation, ECM, and fibroblast programmes (Sung and Lee, 2024; Barrett et al., 2024; Gonzalez Castro et al., 2025; Huo et al., 2024; Xu et al., 2022; Dong et al., 2025). Cohorts are small, often surgically selected, and lack matched longitudinal immunotherapy outcomes. VS provides a descriptive contrast in a benign nerve tumour but is not a validated immune-normalisation template for HNSCC.

## CAF-mediated therapeutic resistance

Defined CAF states can contribute to pre-existing or acquired treatment resistance through matrix, paracrine, metabolic, and immune mechanisms (Chen et al., 2026; Straussman et al., 2012; Tavana and Luker, 2023). Mechanistic support is uneven across treatment modalities and fibroblast states, and many proposed pathways remain extrapolations from non-HNSCC systems.

A dense ECM can raise interstitial pressure and restrict drug distribution, although this mechanism is best established in highly desmoplastic pancreatic models (Heldin et al., 2004; Olive et al., 2009; Provenzano et al., 2012). Those experiments demonstrate that stromal or matrix perturbation can improve drug delivery in pancreatic cancer, but neither quantify this effect in HNSCC nor justify its direct extrapolation. In HNSCC, ECM-rich invasive fronts and nodal deposits are plausible local barriers; direct patient-level measurements linking CAF density, intratumoural drug concentration, and outcome remain limited.

NF-κB signalling can induce anti-apoptotic programmes, including Bcl-2 and survivin (Mao et al., 2025), but this general mechanism does not by itself establish that CAF-derived IL-6 is causal in HNSCC chemotherapy resistance. Reviews and primary studies have proposed midkine-, interleukin-, ANRIL-, autophagy-, and exosomal-microRNA mechanisms of CAF-associated drug resistance across tumour types (Wu et al., 2021; Meng et al., 2024; Zhao et al., 2023; Tassinari and Gandellini, 2021). A separate mechanistic study linked ANRIL to ATR stabilisation and homologous-recombination repair (Liu et al., 2021), but it did not establish a CAF-to-HNSCC pathway. These remain candidate mechanisms pending HNSCC-specific fibroblast perturbation and treatment-response data.

Autophagy may contribute to CAF-associated chemoresistance by sustaining tumour-cell homeostasis during cytotoxic stress and facilitating post-treatment recovery. In an NPC model, CAF-derived exosomes promoted cisplatin resistance through Rheb/mTOR-associated autophagy (Meng et al., 2024). More broadly, autophagy has been implicated in treatment tolerance and recovery after cytotoxic stress (Meng et al., 2024; White, 2012), and autophagy inhibitors have been evaluated in combination with anticancer therapy (Levy et al., 2017). No CAF-specific autophagy target has been established in HNSCC; translation requires evidence that a defined HNSCC fibroblast state induces targetable autophagic flux in tumour cells.

Resistance to radiotherapy: ionising radiation affects both tumour and stromal cells. More broadly, post-radiotherapy stromal, vascular, and immune changes can support resistance or recurrence across tumour types (Barker et al., 2015). Patient-derived HNSCC CAFs can enter senescence after *ex vivo* irradiation while retaining protumourigenic properties (Raaijmakers et al., 2026), consistent with a senescence-associated secretory phenotype that includes cytokines, growth factors, and proteases (Coppe et al., 2010). In HNSCC co-culture models, fibroblast-secreted IL-6 increased tumour-cell radioresistance, although signalling dependencies differed between HPV-positive and HPV-negative cells. In OSCC models, IL-6 activated STAT3 and NRF2, reduced radiation-associated oxidative stress and DNA damage, and increased clonogenic survival; separate experiments showed that irradiated oral CAFs reduced tumour-cell DNA damage and facilitated survival after irradiation. Collectively, these studies support several model-specific stromal routes to radioresistance rather than a single established CAF–IL-6–STAT3–NRF2 mechanism. Their relative contributions in patients and value as prospective biomarkers remain uncertain (Li et al., 2025; Matsuoka et al., 2016; Liu et al., 2023).

mTOR signalling can confer resistance to targeted agents (Guri and Hall, 2016), but this general mechanism does not establish that CAFs induce a reversible quiescent state after radiotherapy in HNSCC. In a patient-derived HNSCC co-culture model, CAFs supported tumour-cell colonisation and post-irradiation viability without demonstrating increased intrinsic radiosensitivity (Federspiel et al., 2025b). CAF-rich and hypoxic regions may overlap clinically, and hypoxic tumour cells are relatively radioresistant (Li X. et al., 2024). Across non-HNSCC models and reviews, CAF-associated vascular abnormalities and stromal oxygen consumption have been proposed to aggravate hypoxia (Jain, 2005; Zhang et al., 2023). Their magnitude and causal contribution in patients with HNSCC remain unclear.

Resistance to targeted therapy: in preclinical HNSCC models, TGF-β-activated CAFs limited cetuximab efficacy, and stromal TGF-β inhibition improved tumour control (Yegodayev et al., 2020). A subsequent study reported cetuximab-induced tumour-cell TGF-β2, fibroblast lipid secretion, and fatty-acid transfer that supported residual-cell survival; combined metabolic or TGF-β-directed interventions restored sensitivity in preclinical models (Vigneron et al., 2026). The TGF-β2–lipid-transfer feedback has been defined only in preclinical models, and neither study establishes a standard clinical combination.

Systemic therapy for NF2-related VS can include bevacizumab in selected patients (Plotkin et al., 2009; Screnci et al., 2024). Variable response or later growth does not by itself implicate the fibroblast compartment. No study has yet shown that VS fibroblast abundance, fibrosis, or a defined paracrine programme causes bevacizumab or radiotherapy resistance. Establishing such mechanisms will require treatment-linked tissue, spatial biomarkers, and functional co-culture or *in vivo* models rather than extrapolation from HNSCC mechanisms.

Resistance to immunotherapy: CAF-derived matrix, TGF-β programmes, and state-specific inhibitory ligands can contribute to T-cell exclusion or dysfunction (Li C. et al., 2024; Krishnamurty et al., 2022; Dominguez et al., 2020; Mariathasan et al., 2018). However, association with a CAF signature does not prove that CAFs are the dominant cause of checkpoint-inhibitor failure. In HNSCC, immune context differs by HPV status, anatomical site, prior therapy, tumour genomics, and myeloid composition (Jenkins et al., 2025; Li C. et al., 2024; Obra et al., 2022; Liu et al., 2025). Prospective evaluation of these mechanisms requires CAF-state biomarkers, spatial immune readouts, and treatment-linked biopsies.

Combined TGF-β and checkpoint blockade has a strong preclinical rationale, but clinical trials have not consistently reproduced the model efficacy (Metropulos et al., 2022). In a randomised double-blind phase II study of 55 patients with recurrent/metastatic, CAF-rich HNSCC, the NOX1/4 inhibitor setanaxib plus pembrolizumab did not improve the primary endpoint of best percentage change in tumour size; exploratory PFS, OS, and intratumoural CD8^+^ findings favoured the combination using 80% confidence intervals and a prespecified nominal p-value threshold of 0.20 (Fayette et al., 2024). The phase II estimates are hypothesis-generating, sponsor-funded, and require confirmatory trials. Losartan is an angiotensin-receptor blocker with antifibrotic effects—not an antifibrinolytic agent—and the HNSCC combination study has no posted efficacy results (Wan et al., 2026; Losartan, 2026).

CAF-derived signals may create tumour-cell dependencies that vary across experimental and disease settings. Sustained mitogenic or survival signalling, for example, may increase reliance on MEK–ERK or anti-apoptotic pathways and thereby create a candidate synthetic-lethal vulnerability (El Herch et al., 2024). Such dependencies remain model-specific: co-culture or pathway activation does not establish therapeutic selectivity in patients, and inhibition may also affect immune or normal stromal cells. Assigning such vulnerabilities to CAF biology in HNSCC requires genetic or pharmacological perturbation, confirmation in patient-derived models, and biomarker-linked clinical testing.

## Therapeutic strategies targeting CAFs

CAF-directed therapy remains investigational in HNSCC. Strategies include blocking activation pathways, inhibiting state-specific signals, altering matrix mechanics, selectively depleting defined populations, or reprogramming fibroblast states (Sahai et al., 2020; Tavana and Luker, 2023; Boeker and Kalluri, 2025). The therapeutic window is constrained by fibroblast contributions to tissue integrity and immune organisation and by CAF states that may restrain tumour growth or support antitumour immunity (Hutton et al., 2021; Ozdemir et al., 2014; Rhim et al., 2014). No universal hierarchy among depletion, normalisation, and pathway blockade has been established. The evidence base is stratified by clinical stage and disease setting (Table 3).

**TABLE 3:** CAF-directed therapeutic strategies and level of evidence.

Strategy and agent/target	Disease and evidence level	Key result	Interpretation and status
NOX1/4–myCAF modulation: Setanaxib + pembrolizumab	HNSCC; randomised phase II, n = 55 [107]	Primary tumour-size endpoint negative; exploratory PFS/OS and CD8 signals	Hypothesis-generating; confirmatory study needed
TGF-β/PD-L1 trap: Bintrafusp alfa	HNSCC; phase I, n = 32 [115]	IRC ORR 13%; grade 3 TRAEs 34%	Early safety/activity; no randomised efficacy conclusion
TGF-β/PD-L1 trap: Bintrafusp alfa	HNSCC; neoadjuvant, n = 14 [116]	Feasible; no surgical delay	Small non-randomised feasibility study
TGF-β/SMAD: Halofuginone	OSCC; preclinical (Wang et al., 2022)	Reduced CAF activity and nodal metastasis	Not clinical evidence
NOX4: Genetic/pharmacological inhibition	Preclinical CAF-rich models (Ford et al., 2020; Hanley et al., 2018)	Reversed myCAF phenotype; improved CD8 access/ICI response	Mechanistic rationale for biomarker-led trials
Antifibrotic: Losartan + pembrolizumab + SBRT	HNSCC; NCT06211335 [109]	No posted efficacy results	Ongoing; efficacy remains undetermined
Selective depletion: FAP-directed approaches	Non-HNSCC preclinical (Tran et al., 2013; Loeffler et al., 2006)	Antitumour activity but normal-stroma toxicity	Requires tumour-restricted target
Stromal reprogramming: ATRA/VDR agonism	PDAC preclinical/phase I (Froeling et al., 2011; Kocher et al., 2020; Sherman et al., 2014)	Quiescent-state and feasibility signals	Evidence from other tumour types only
CXCL12–CXCR4: CXCR4 antagonism + PD-1	PDAC preclinical/phase II (Feig et al., 2013; Bever et al., 2025)	T-cell recruitment with compensatory myeloid effects	Biomarker and combination strategy unresolved

TGF-β-directed approaches illustrate both rationale and limitation. Halofuginone reduced CAF activity and nodal metastasis in an oral squamous cell carcinoma model (Wang et al., 2022). Bintrafusp alfa, a bifunctional TGF-β trap/PD-L1 antibody, produced an independently reviewed objective response rate of 13% in a 32-patient phase I HNSCC cohort, with grade 3 treatment-related adverse events in 34% (Cho et al., 2020); a separate 14-patient neoadjuvant study showed feasibility without surgical delay (Redman et al., 2022). These small non-randomised studies cannot establish efficacy, and broad TGF-β blockade may affect immune regulation and normal tissue repair. Biomarker-defined combinations and pharmacodynamic confirmation are needed.

Direct CAF depletion has produced divergent outcomes. FAP-directed cells or vaccines reduced stroma and improved therapy in several mouse models, but FAP is also expressed by normal stromal populations and FAP-targeted T cells caused cachexia and bone-marrow toxicity in mice (Tran et al., 2013; Loeffler et al., 2006). In pancreatic cancer, genetic or pharmacological depletion of α-SMA-positive fibroblasts accelerated disease and increased immunosuppression (Ozdemir et al., 2014; Rhim et al., 2014). Although these findings derive from non-HNSCC models, they caution against indiscriminate depletion. Selective targeting requires a stable, tumour-restricted marker linked to a functionally validated pathogenic fibroblast state.

All-trans retinoic acid and vitamin-D-receptor agonism induced quiescent stromal programmes in pancreatic models, and ATRA reached a phase I pancreatic study (Froeling et al., 2011; Kocher et al., 2020; Sherman et al., 2014). Pancreatic data do not show that HNSCC CAFs can be converted to a VS-like state. Clinical evaluation in HNSCC would require pretreatment state selection, paired biopsies demonstrating a defined phenotypic shift, and evidence that normalisation does not expand an alternative immunosuppressive population.

Blocking paracrine signals may be more selective than eliminating fibroblasts, but most examples are supported principally by studies in other tumour types. CXCL12–CXCR4 blockade enhanced checkpoint therapy in pancreatic models and produced complex immune changes in a pancreatic phase II study (Feig et al., 2013; Bever et al., 2025). HGF–MET and IL-6 pathways are supported by mechanistic studies, including HNSCC radioresistance data for fibroblast-derived IL-6 (Straussman et al., 2012; Li et al., 2025). Translation requires confirmation that the targeted ligand is produced by the relevant HNSCC fibroblast state and is not redundant with parallel signals.

Matrix-directed approaches also require caution. LOX inhibition can reduce collagen crosslinking, while hyaluronidase strategies were developed primarily for hyaluronan-rich pancreatic tumours (Cetin et al., 2024; Wong et al., 2017). Matrix removal may improve penetration but can alter vascular integrity, immune trafficking, and physical restraint. Clinical development in HNSCC will require direct measurements of matrix composition and mechanics rather than patient selection based solely on bulk CAF abundance.

Metabolic and autophagy-directed strategies are supported by preclinical studies, including secretory autophagy in head and neck CAFs (New et al., 2017). Shikonin-mediated suppression of CAF mitochondrial support was demonstrated in triple-negative breast cancer (Fan et al., 2024), whereas proposed use of glycolysis, mitochondrial, or autophagy inhibitors in HNSCC remains non-selective and unproven. Because these pathways are shared with immune and epithelial cells, cell-type-specific delivery and pharmacodynamic biomarkers are essential.

Drug repurposing requires explicit stratification by evidence level. Losartan has reduced collagen production and improved drug distribution in preclinical tumours and has been evaluated clinically in pancreatic cancer (Diop-Frimpong et al., 2011; Murphy et al., 2019); the HNSCC trial is ongoing and currently has no posted results (Losartan, 2026). Setanaxib provides the most direct randomised HNSCC test of a CAF-linked mechanism to date, but its negative primary endpoint and exploratory statistical design require cautious interpretation (Fayette et al., 2024). NOX4 inhibition reversed myofibroblast phenotypes and restored immune infiltration in preclinical models, including head-and-neck-tumour tissue analyses (Ford et al., 2020; Hanley et al., 2018).

Therapeutic objectives should be defined by the pathogenic fibroblast state and clinical context rather than by an *a priori* preference for depletion or normalisation. A biomarker-led trial design should include (i) a reproducible CAF-state and spatial biomarker; (ii) evidence that the state contributes causally to the target phenotype and is pharmacologically tractable; (iii) paired tissue pharmacodynamics; (iv) immune and matrix safety readouts; and (v) a standard-of-care combination selected for the predicted resistance mechanism. This approach permits suppression of a defined tumour-promoting programme while monitoring preservation of homeostatic stromal functions (Sahai et al., 2020; Boeker and Kalluri, 2025; Jain, 2013).

VS can inform this programme only as a discovery comparator. Its value should be tested through matched, cell-type-resolved analyses of HNSCC CAFs, normal oral fibroblasts, VS fibroblasts, and normal nerve-associated fibroblasts under standardised culture and spatial conditions. Candidate differences require perturbational testing in organotypic HNSCC and VS models. Without these controls, a pathway observed in VS cannot be classified as protective, and its absence from a small VS dataset cannot establish HNSCC specificity.

## Conclusion

CAFs are heterogeneous and dynamic components of the HNSCC microenvironment. Their effects arise from state- and location-dependent interactions with tumour, immune, vascular, and matrix compartments; they cannot be reduced to a single uniformly tumour-promoting population. VS may serve as a benign stromal comparator, but differences in lineage, anatomy, oncogenic drivers, and immune context preclude biological equivalence or attribution of its non-metastatic behaviour to fibroblasts. The strongest HNSCC evidence concerns defined fibroblast states associated with invasion, immune exclusion, or immune support, whereas most therapeutic mechanisms remain preclinical. Early trials of TGF-β/PD-L1 and NOX1/4-directed combinations provide feasibility data and hypothesis-generating efficacy signals but do not define a standard of care. Progress will require spatially resolved state definitions, functional perturbation, paired treatment biopsies, and biomarkers that preserve homeostatic or antitumour fibroblast functions. Future studies should connect spatially defined CAF states to causal functions, pharmacodynamic responses, and clinical outcomes before stromal interventions are advanced into routine treatment.

## Glossary

ANG1/ANG2: Angiopoietin 1/2
ANRIL: Antisense non-coding RNA in the INK4 locus
apCAFs: Antigen-presenting CAFs
ATRA: All-trans retinoic acid
Bcl-2: B-cell lymphoma two
Bcl-xL: B-cell lymphoma-extra large
BMPs: Bone morphogenetic proteins
CAF: Cancer-associated fibroblast
CAFs: Cancer-associated fibroblasts
CAR-T: Chimeric antigen receptor T cells
CCL5: Chemokine (C-C motif) ligand five
CCL21: Chemokine (C-C motif) ligand 21
COX-2: Cyclooxygenase-2
CTGF: Connective tissue growth factor
CXCL9: Chemokine (C-X-C motif) ligand nine
CXCL10: Chemokine (C-X-C motif) ligand 10
CXCL12: Chemokine (C-X-C motif) ligand 12
CXCL13: Chemokine (C-X-C motif) ligand 13
CYR61: Cysteine-rich angiogenic inducer 61
DC: Dendritic cell
ECM: Extracellular matrix
EGF: Epidermal growth factor
ecmCAFs: ECM-related CAFs
EMT: Epithelial–mesenchymal transition
EndMT: Endothelial-to-mesenchymal transition
FAK: Focal adhesion kinase
FAP: Fibroblast activation protein
FGF2: Fibroblast growth factor 2
FRC: Fibroblastic reticular cell
HGF: Hepatocyte growth factor
Hippo–YAP/TAZ: Hippo–YAP/TAZ pathway
HN: Head and neck cancers
HNSCC: Head and neck squamous cell carcinoma
HPV: Human papillomavirus
iCAFs: Inflammatory CAFs
ICIs: Immune checkpoint inhibitors
IGF1: Insulin-like growth factor 1
IL-2: Interleukin-2
IL-6: Interleukin-6
IL-8: Interleukin-8
IL-10: Interleukin-10
IL-12: Interleukin-12
IRC: Independent review committee
JAK2/JAK3: Janus kinase 2/3
LIF: Leukaemia inhibitory factor
lncRNA: Long non-coding RNA
LOX: Lysyl oxidase
LRRC15: Leucine-rich repeat-containing protein 15
Mcl-1: Myeloid cell leukemia-1
MDSCs: Myeloid-derived suppressor cells
MHC: Major histocompatibility complex
MMPs: Matrix metalloproteinases
MSCs: Mesenchymal stem cells
myCAFs: Myofibroblastic CAFs
NF2: Neurofibromatosis type 2 (Merlin)
NFs: Normal fibroblasts
NK cells: Natural killer cells
NOX1/4: NADPH oxidase 1/4
NPC: Nasopharyngeal carcinoma
Nrf2: Nuclear factor erythroid 2-related factor 2
ORR: Objective response rate
OS: Overall survival
OSCC: Oral squamous cell carcinoma
OXPHOS: Oxidative phosphorylation
PD-L1: Programmed death-ligand one
PD-L2: Programmed death-ligand two
PDGF-BB: Platelet-derived growth factor-BB
PEGPH20: PEGylated hyaluronidase
PFS: Progression-free survival
**PGE**_**2**_: Prostaglandin E_2_
PI3K/AKT/mTOR: PI3K/AKT/mTOR pathway
RTKs: Receptor tyrosine kinases
SASP: Senescence-associated secretory phenotype
SBRT: Stereotactic body radiotherapy
SDF-1: Stromal cell-derived factor 1 (CXCL12)
SOCS3: Suppressor of cytokine signaling three
SPP1: Osteopontin (secreted phosphoprotein 1)
STAT3: Signal transducer and activator of transcription three
TAM: Tumour-associated macrophage
TAZ: Transcriptional co-activator with PDZ-binding motif
TGF-β: Transforming growth factor-β
Th1: T helper one cells
TME: Tumour microenvironment
TRAEs: Treatment-related adverse events
Treg: Regulatory T cell
VEGF: Vascular endothelial growth factor
VS: Vestibular schwannoma
WNT3A: Wnt family member 3 A
Wnt/β-catenin: Wnt/β-catenin pathway
YAP: Yes-associated protein
α-SMA: α-smooth muscle actin
